# Supplementary material for: Quantitative trait loci mapping for salt tolerance-related traits during the germination stage of wheat
Source: PLoS One. 2025 Apr 2;20(4):e0319411. doi: 10.1371/journal.pone.0319411 (PMC11964244; doi:10.1371/journal.pone.0319411)
Supplement: S1 Table — (DOCX) [file pone.0319411.s001.docx]

**S1 Table.** 1**96 varieties of wheat information**

| **No.** | **Variety Name** |
| --- | --- |
| 1 | Yannong 0428 |
| 2 | New Variety 13 |
| 3 | Xingmai 6 |
| 4 | 7DL 7Ag |
| 5 | Jinmai 70 |
| 6 | Shixin 828 |
| 7 | Womai U876 |
| 8 | Jinmai 59 |
| 9 | Tang 08-4021 |
| 10 | Yunhei 161 |
| 11 | Donghei 1 |
| 12 | Keyi 10-6014 |
| 13 | Baomai 3 |
| 14 | Linzi 217 |
| 15 | Keyi 11-6072 |
| 16 | Taihang 2008 Selection |
| 17 | Jingdong 8 |
| 18 | Nongda 399 |
| 19 | Heng 05-6607 |
| 20 | Cangmai 2016-8 |
| 21 | Jinhai 13294 |
| 22 | Demai 1201 |
| 23 | Jin 07214 |
| 24 | Baomai 9 |
| 25 | 10RH13 |
| 26 | Jingdong 20 |
| 27 | Sui Xuan Hei |
| 28 | Heng 5229 |
| 29 | Ji 6358 |
| 30 | Qiushuo 996 |
| 31 | Shannong 6-8-6 |
| 32 | Jingdong 10 |
| 33 | Luyuan 502 |
| 34 | Jin Nong 5 |
| 35 | Jinmai 57 |
| 36 | Fuzhao 5066 |
| 37 | Zhongzhong Mai 17 |
| 38 | Shinong 086 |
| 39 | Baofeng 104 |
| 40 | Fumai 6 |
| 41 | Heng 95 Guan 26 |
| 42 | Zhongmai 1062 |
| 43 | Lun Xuan 901 |
| 44 | Kenong 9204 |
| 45 | Jimai 585 |
| 46 | Jinmai 33 |
| 47 | Shinxin 811 |
| 48 | Zhongxin 5199 |
| 49 | Heng 71-3 |
| 50 | Linfen 7061 |
| 51 | Jingdong 8 |
| 52 | Jinmai 66 |
| 53 | Shannong 6-8-2 |
| 54 | Tang 10-4303 |
| 55 | New Variety 18 |
| 56 | Jimai 30 (Ji 5418) |
| 57 | Tang 08-4322 |
| 58 | Heng H1835 |
| 59 | Ji Shi 02-1 |
| 60 | Tianhe 9 |
| 61 | Henong 6049 |
| 62 | Shiao 1 |
| 63 | Western Agriculture 979 |
| 64 | Shinxin 733 |
| 65 | Liangxing 66 |
| 66 | Ji 5265 |
| 67 | Ji Zimai 20 |
| 68 | Jinhai 14219 |
| 69 | Shimai 16 |
| 70 | Zhenghan 36 |
| 71 | Jinghua 1 |
| 72 | Hangmai 802 |
| 73 | Ji Fu 85012 |
| 74 | Heng Mixed 4 |
| 75 | Handan Mai 12 (4015) |
| 76 | Heng 4371 |
| 77 | Jin Boshi 731 |
| 78 | JF416 |
| 79 | Luo 13122 |
| 80 | Ji Mai 220 |
| 81 | RS1212 |
| 82 | Jin Rui 20 |
| 83 | Cangmai 6005 |
| 84 | Handake 4161 |
| 85 | Jing 9428 |
| 86 | Heng 7228 |
| 87 | Cangmai 119 |
| 88 | Hengshui 4 |
| 89 | Tiegan Mai Wang |
| 90 | Cangmai 201830 |
| 91 | Yuanfeng 175 |
| 92 | Shixin 633 |
| 93 | Shinong 958 |
| 94 | Shi 4366 |
| 95 | Taimai 198 |
| 96 | Ji Zimai 17 |
| 97 | Hongmai 15-1 |
| 98 | Jingdong 13 |
| 99 | Fumai 5 |
| 100 | Henong 628 |
| 101 | Jidong 3097 |
| 102 | Shannong 08-29 |
| 103 | Shijiazhuang 11 |
| 104 | Ji Purple 439 |
| 105 | Youmai 66 |
| 106 | Jingdong 18 |
| 107 | New Variety 17 |
| 108 | Jinmai 63 |
| 109 | Wotu 808 |
| 110 | Tianhe 6 |
| 111 | Liangxing 77 |
| 112 | Shi 4185 |
| 113 | Zhongxin Mai 78 |
| 114 | Baomai 12-6 |
| 115 | Cangmai 6004 |
| 116 | Zhongmai 122 |
| 117 | Jingdong 24 |
| 118 | Boma 8450 |
| 119 | Daman 77 |
| 120 | Taihang 2008 |
| 121 | Gao You 9415 |
| 122 | Tang 08-S6 |
| 123 | Shandong Purple Wheat 1 |
| 124 | Heng 136 |
| 125 | Zhongmai 175 |
| 126 | Henong 7008 |
| 127 | Shi 15-5608 |
| 128 | Baomai Pin 8 |
| 129 | Heng 6632 |
| 130 | Jingdong 17 |
| 131 | Tongshuai 113 |
| 132 | Heng 4399 |
| 133 | Hangmai 247 |
| 134 | Nongda 5181 |
| 135 | Shannong 7-9-3 |
| 136 | Jie Mai 20 |
| 137 | Nongda 136 |
| 138 | Xiaoyan 81 |
| 139 | Ruika 288 |
| 140 | Henong 825 |
| 141 | YTXYMH Cooperative Green |
| 142 | Zhongxin Mai 48 |
| 143 | Denghai 202 |
| 144 | Le 639 |
| 145 | Bao 6834 |
| 146 | Handan 06-5170 |
| 147 | Jimai 403 |
| 148 | Green Treasure |
| 149 | Jingdong 11 |
| 150 | Jingdong 22 |
| 151 | Shijiazhuang 8 |
| 152 | Baomai 8 |
| 153 | Cangmai 2018-3 |
| 154 | Shannong J 25-229 |
| 155 | Handan Early 1 |
| 156 | Heng 05 Guan 33 |
| 157 | Ji Zimai 3 |
| 158 | Shiyou 17 |
| 159 | Jimai 518 |
| 160 | Liangxing 99 |
| 161 | Aimai 28 |
| 162 | Tang 10-4102 |
| 163 | Handan 6172 |
| 164 | Jinghua 9 |
| 165 | 17AS233 |
| 166 | Henong 2552 |
| 167 | Shi H083-366 |
| 168 | Gao You 9409 |
| 169 | Shimai 15 |
| 170 | Nongda 211 |
| 171 | Jinhai 639 |
| 172 | Tang 09-4450 |
| 173 | Jimai 325 |
| 174 | Gao You 2018 |
| 175 | Heng 05-4444 |
| 176 | Handan 5316 |
| 177 | Huangjin Mai 567 |
| 178 | Henong 8060 |
| 179 | Tang 09-4132 |
| 180 | Qiushuo 882 |
| 181 | Nongda 3432 |
| 182 | Jinghua 11 |
| 183 | Jing 9843 |
| 184 | Nongda 212 |
| 185 | Jingdong 23 |
| 186 | Tang 10-4409 |
| 187 | Lun Xuan 103 |
| 188 | Cangmai 2017-20 |
| 189 | Henong 05 (9) 7-11-4-15 |
| 190 | Tang 09-418 |
| 191 | Nongda 1108 |
| 192 | Zhongmai 629 |
| 193 | Handan Mai 9 (9587) |
| 194 | Luo 13012 |
| 195 | Tang 10-4018 |
| 196 | Keyi 5214 |
