# Supplementary material for: Quantitative trait loci mapping for salt tolerance-related traits during the germination stage of wheat
Source: PLoS One. 2025 Apr 2;20(4):e0319411. doi: 10.1371/journal.pone.0319411 (PMC11964244; doi:10.1371/journal.pone.0319411)
Supplement: Attachment — Submitted filename: Response to Reviewers.docx [file pone.0319411.s004.docx]

**Part 1 Revision comments from Aimin Zhang, Ph.D., Academic Editor.**

1. Please ensure that your manuscript meets PLOS ONE's style requirements, including those for file naming. The PLOS ONE style templates can be found at

https://journals.plos.org/plosone/s/file?id=wjVg/PLOSOne_formatting_sample_main_body.pdf and

https://journals.plos.org/plosone/s/file?id=ba62/PLOSOne_formatting_sample_title_authors_affiliations.pdf

Response: Thank you very much. I have made the revisions according to the templates.

2. In your Methods section, please provide additional information regarding the permits you obtained for the work. Please ensure you have included the full name of the authority that approved the field site access and, if no permits were required, a brief statement explaining why.

Response: The research activities and the experimental materials and data used in this study were authorized by the Wheat Research Institute Laboratory of the Tangshan Academy of Agricultural Sciences. This has been supplemented in the materials section of the article.

3. Please note that PLOS ONE has specific guidelines on code sharing for submissions in which author-generated code underpins the findings in the manuscript. In these cases, we expect all author-generated code to be made available without restrictions upon publication of the work. Please review our guidelines at https://journals.plos.org/plosone/s/materials-and-software-sharing#loc-sharing-code and ensure that your code is shared in a way that follows best practice and facilitates reproducibility and reuse.

Response: This article does not include relevant code.

4. Thank you for stating the following financial disclosure:

The work was funded by a number of projects, including the Hebei Province Major Science and Technology Support Program Project: Innovation, Integration, and Application of Technology Models for Capacity Enhancement in Coastal Saline-Alkali Areas (242N6401Z);Hebei Province Modern Agricultural Industry Technology System Drought-Alkali Wheat Innovation Team-Coastal Saline-alkali Crop Comprehensive Ex-periment and Promotion Station (HBCT2024030404); Tangshan City Science and Tech-nology Program Projects (23150201A, 23150204A, 24150204C); Hebei Province Modern Agricultural Industry Technology System Wheat Innovation Team — Tangshan Rice Ex-perimental Station (HBCT2024010408).

*Please state what role the funders took in the study. If the funders had no role, please state: ""The funders had no role in study design, data collection and analysis, decision to publish, or preparation of the manuscript.""*

*If this statement is not correct you must amend it as needed.*

*Please include this amended Role of Funder statement in your cover letter; we will change the online submission form on your behalf.*

Response: The funders had no role in study design, data collection and analysis, decision to publish, or preparation of the manuscript. I have stated this in the cover letter as required.

*5. Please include a copy of Table 4 which you refer to in your text on page 38.*

Response: I apologize for that; it’s a mistake. It should be Table 3, as there is no Table 4. I have corrected it.

*6. Please include captions for your Supporting Information files at the end of your manuscript, and update any in-text citations to match accordingly. Please see our Supporting Information guidelines for more information: http://journals.plos.org/plosone/s/supporting-information.*

Response: Supporting Information has been added as required

**Part 2 Revision comments from Reviewer 1.**

Soil salinization can affect the growth and development of wheat, and the QTL for salt stress tolerance is of great value in the genetic improvement of wheat stress tolerance in molecular breeding programs. This study reported 22 QTLs significantly associated with salt tolerance in wheat germination stage, and 175 annotated genes were discovered in the six major QTLs. This is a useful study to some extent. My major concerns are as follows:

1. Figure 1 makes no sense and could be removed from the text.

Response: Thank you very much. The title above the Figure has been removed.

2. The broad-sense heritability of the corresponding phenotypic trait should be supplemented, if possible.

Response: I have added broad-sense heritability in the last row at the bottom of Table 1.

3. Figure 3, the title above the Figure should be removed, as the corresponding information can be seen in the legend.

Response: The title above the figure has been removed.

4. Figure 4, the cultivar names of the corresponding representative samples should be supplemented, if possible.

Response: I have indicated the names of the respective varieties in the legend section of Figure 4. T(tolerance, Nongda 399), MT(medium tolerance, Liangxing 66), S(sensitivity, Ruika 288) and HS(high sensitivity, Jinghua 11).

5. Only D-value was used for GWAS. I suppose to perform GWAS for both D-values and the phenotypic traits under different treatments. Please also compare their mapping position and discover more useful information.

Response: Thank you. I have added the relevant experimental results, figures, and discussion. In the association analysis between traits and significant QTL loci, we found that all traits except for GR and RSIR successfully associated with their corresponding QTLs. Specifically, the GP trait was particularly prominent, identifying the highest number of QTLs, totaling 36, among which GP_0 and GP_1 were associated with 2 QTLs each, while RGP had the most, with 32 QTLs. Next, D_Value was associated with 32 QTLs. The RN trait was associated with 28 QTLs, with further subdivision revealing that RN_0, RN_1, and RRN were associated with 1, 20, and 7 QTLs, respectively. In contrast, the number of QTLs associated with the RFW trait was relatively lower, with only RFW_1 linking to 4 QTLs. As for the RL trait, there were a total of 3 QTLs, with RL_1 containing 1 and RRL containing 2. Additionally, the SFW and SL traits were also associated with a certain number of QTLs under control treatment conditions, with SFW_0 associated with 3 QTL loci and SL_0 associated with 2 QTLs. Please refer to the results and discussion in the revised manuscript for more supplementary information.

6. Please compare the QTL with that of previous studies based on their physical position or common markers, and determine the novel QTL that were firstly reported in this study. In addition, are there common QTL for yielding potential that co-located with the salt stress tolerance?

Response: I have added references to the research results of Akram, Asif, Guo, Javid, Khan, Oyiga, Pasam, Amin, and Masoudi. Please refer to the discussion section of the article for details.

7. Overall, the English writing, the whole chart/table layout should be improved.

Response: Thank you. The English writing, the whole chart/table layout has been modified and improved.

**Part 3 Revision comments from Reviewer 2.**

This manuscript “Quantitative Trait Loci Mapping for Salt Tolerance-Related Traits during the Germination Stage of Wheat” by Song et al. This study investigates the genetic basis of salt tolerance in wheat during the germination stage by analyzing 196 wheat varieties from China. The study evaluated salt tolerance traits using principal component analysis (PCA) and categorized the varieties into five tolerance levels. A genome-wide association study (GWAS) with a 16Kb SNP chip identified 22 significant QTLs across six chromosomes (1B, 2A, 3B, 4B, 6A, and 7D), and gene annotation revealed 11 potential candidate genes involved in stress response mechanisms. The findings provide a theoretical foundation for cloning salt-tolerance genes and breeding salt-tolerant wheat varieties to address agricultural challenges in saline-alkaline soils. However, certain aspects require refinement before this can be considered ready for publication:

Comment 1. In the introduction, Please simplify repetitive phrasing by removing “Province” after each name, and replacing “measuring” with “covering” for better word choice.

Response: Thank you, I have removed ‘Province’ and replaced ‘measuring’ with ‘covering’.

Comment 2. In the discussion, improving influence by rephrasing “cloning genes related to salt tolerance” as “cloning salt tolerance-related genes”.

Response: I have made the modification to the discussion by rephrasing ‘cloning genes related to salt tolerance’ as ‘cloning salt tolerance-related genes’

Comment 3. Add units to the header of table 1 (For example: "root length (cm)").

Response: I have added units to the header of Table 1

Comment 4. Add references to paragraph 3 on page 25.

Response: I have added references to paragraph 3 on page 25

Comment 5. It is recommended that a figure legend and table legend be added.

Response: I have added a figure legend and a table legend as recommended.

Comment 6. Note the format of the figures and tables.

Response: Thank you. I have noted the format of the figures and tables.

Comment 7. Please make additional careful revisions to the formatting of the manuscript.

Response: Thank you. I have made additional careful revisions to the formatting of the manuscript.
